# Mesenchymal stromal cells induce inhibitory effects on hepatocellular carcinoma through various signaling pathways

**DOI:** 10.1186/s12935-019-1038-0

**Published:** 2019-12-05

**Authors:** Jafar Ai, Neda Ketabchi, Javad Verdi, Nematollah Gheibi, Hossein Khadem Haghighian, Maria Kavianpour

**Affiliations:** 1 Department of Tissue Engineering and Applied Cell Sciences, Faculty of Advanced Technologies in Medicine, Tehran University of Medical Sciences, Tehran, Iran; 2 Department of Medical Laboratory Sciences, Maragheh University of Medical Sciences, Maragheh, Iran; 3 Department of Physiology and Medical Physics, Qazvin University of Medical Sciences, Qazvin, Iran; 4 Metabolic Diseases Research Center, Research Institute for Prevention of Non-Communicable Diseases, Qazvin University of Medical Sciences, Qazvin, Iran; 5 Cell‐Based Therapies Research Center, Digestive Disease Research Institute, Tehran University of Medical Sciences, Tehran, Iran

**Keywords:** Mesenchymal stromal cells, Hepatocellular carcinoma, Wnt signaling, Toll like receptor, Nuclear factor-kappa B, JNK pathway

## Abstract

Hepatocellular carcinoma (HCC) is the most prevalent type of malignant liver disease worldwide. Molecular changes in HCC collectively contribute to Wnt/β-catenin, as a tumor proliferative signaling pathway, toll-like receptors (TLRs), nuclear factor-kappa B (NF-κB), as well as the c-Jun NH2-terminal kinase (JNK), predominant signaling pathways linked to the release of tumor-promoting cytokines. It should also be noted that the Hippo signaling pathway plays an important role in organ size control, particularly in promoting tumorigenesis and HCC development. Nowadays, mesenchymal stromal cells (MSCs)-based therapies have been the subject of in vitro, in vivo, and clinical studies for liver such as cirrhosis, liver failure, and HCC. At present, despite the importance of basic molecular pathways of malignancies, limited information has been obtained on this background. Therefore, it can be difficult to determine the true concept of interactions between MSCs and tumor cells. What is known, these cells could migrate toward tumor sites so apply effects via paracrine interaction on HCC cells. For example, one of the inhibitory effects of MSCs is the overexpression of dickkopf-related protein 1 (DKK-1) as an important antagonist of the Wnt signaling pathway. A growing body of research challenging the therapeutic roles of MSCs through the secretion of various trophic factors in HCC. This review illustrates the complex behavior of MSCs and precisely how their inhibitory signals interface with HCC tumor cells.

## Introduction

Hepatocellular carcinoma (HCC) is the most common type of liver malignancy, occurs predominantly in patients with underlying chronic liver disease and cirrhosis [1]. Hepatitis B virus (HBV) and hepatitis C virus (HCV) infections, combined with the advanced stage of liver fibrosis are thought to be the major risk factors for HCC [2].

There are alternative treatments available for this particular type of cancer, including chemotherapy with anti-cancer agents like sorafenib, radiotherapy, and immunotherapy, as well as the surgical resection of tumoral lesions. Ultimately, liver transplantation is an accepted modality of treatment in this background [3]. Although a liver transplant may offer the best chance of survival in patients with end-stage liver disease, the potential complications of this procedure lead to an urgent need to develop new treatment strategies for HCC. Cell therapy research propose some new mechanisms for tissue regeneration that would be used as a suitable replacement alone or in combination with other medications [4, 5].

In recent years, considerable research has been devoted to developing effective HCC treatment options. In particular, mesenchymal stromal cells (MSCs) based therapies have been the subject of in vitro, in vivo, and clinical studies for liver dysfunction management purposes [6]. In addition to the differentiation capacity of MSCs as intrinsic drug stores, they are also able to secrete various trophic factors, affecting a large number of cells through the body [4]. MSCs, also known as fibroblast-like cells possess self-renewal capacity and multiple differentiation abilities to distinct lineages, such as osteocytes, adipocytes, and chondrocytes [7]. Mainly in the bone marrow, they have either been identified in various tissues and organs such as adipose, umbilical cord, kidney, brain, liver, and lung. These organs contain a subpopulation of stem cells as sources of putative MSCs for therapeutic purposes, largely due to findings related to their effectiveness in the treatment of several diseases [8].

The presence of these cells is confirmed mainly by evaluating the cell surface markers, such as CD29, CD51, CD73, CD90, and CD105, as well as the lack of CD45 and CD31 [9]. Still, the evidence of the homogenous population of such cells have not been identified in any research studies. The International Society for Cell and Gene Therapy (ISCT) proposed two criteria for the definition of MSCs based on heterogeneous and nonclonal cell populations, which are a mixture of stem cells with different multipotential properties, committed progenitors, and differentiated cells [10]. Despite the tumor homing nature and induction of cancer cell growth arrest by MSCs [9, 10], still little is known about the signaling molecules and how they work in such cells [11].

Molecular carcinogenesis of HCC could be variable due to either inactivation or loss of tumor suppressor genes such as cyclin-dependent kinase inhibitor 1A (P21, Cip1), tumor protein p53 (TP53), retinoblastoma (RB) and phosphatase and tensin homolog (PTEN) and/or activation of oncogenes including protein kinase B (PKB) and neuroblastoma Ras (N-Ras) [12, 13]. In addition, HCC is strongly correlated with abnormalities of signal transduction network that regulate self-renewal ability, proliferation and differentiation capacity of stem cell, for example, MAPK, mTOR, Notch, and Wnt/β-catenin pathways or another cytokine such as HGF, IGF, VEGF, and PDGF signaling [14, 15]. The homing potential of human MSCs to lesions of Kaposi’s sarcoma and their inhibitory activity on tumor growth by down-regulating the PKB is an example of the therapeutic potency of MSCs co-cultured with animal tumor cells [16]. Hajighasemlou et al. have indicated the huge therapeutic potential of intertumoral injection of MSCs as a cell-based therapy in HCC [17]. Increased MSC migration toward tumor sites is associated with improved biochemical test results due to their growth suppression effect on HCC cells. Nevertheless, we need more experimental data in which signaling pathways are highly involved in this background? In this review, we will discuss the inhibitory effects of MSCs on HCC cell proliferation with the key focus on related signaling transduction network.

### Downregulation of Wnt signaling in HCC

Among the multiple signaling pathways, a significant amount of research has been done studying the Wnt signaling alteration as a common pathogenic basis of HCC [14]. The HCC tumor microenvironment is associated with a hyperactive Wnt signaling pathway, as well as the acquisition of stemness features by tumor cells [18]. It seems likely that the increased expression of downstream target genes is a key step within the pathological consequences of Wnt signaling in HCC cells [19].

**Fig. 1:**
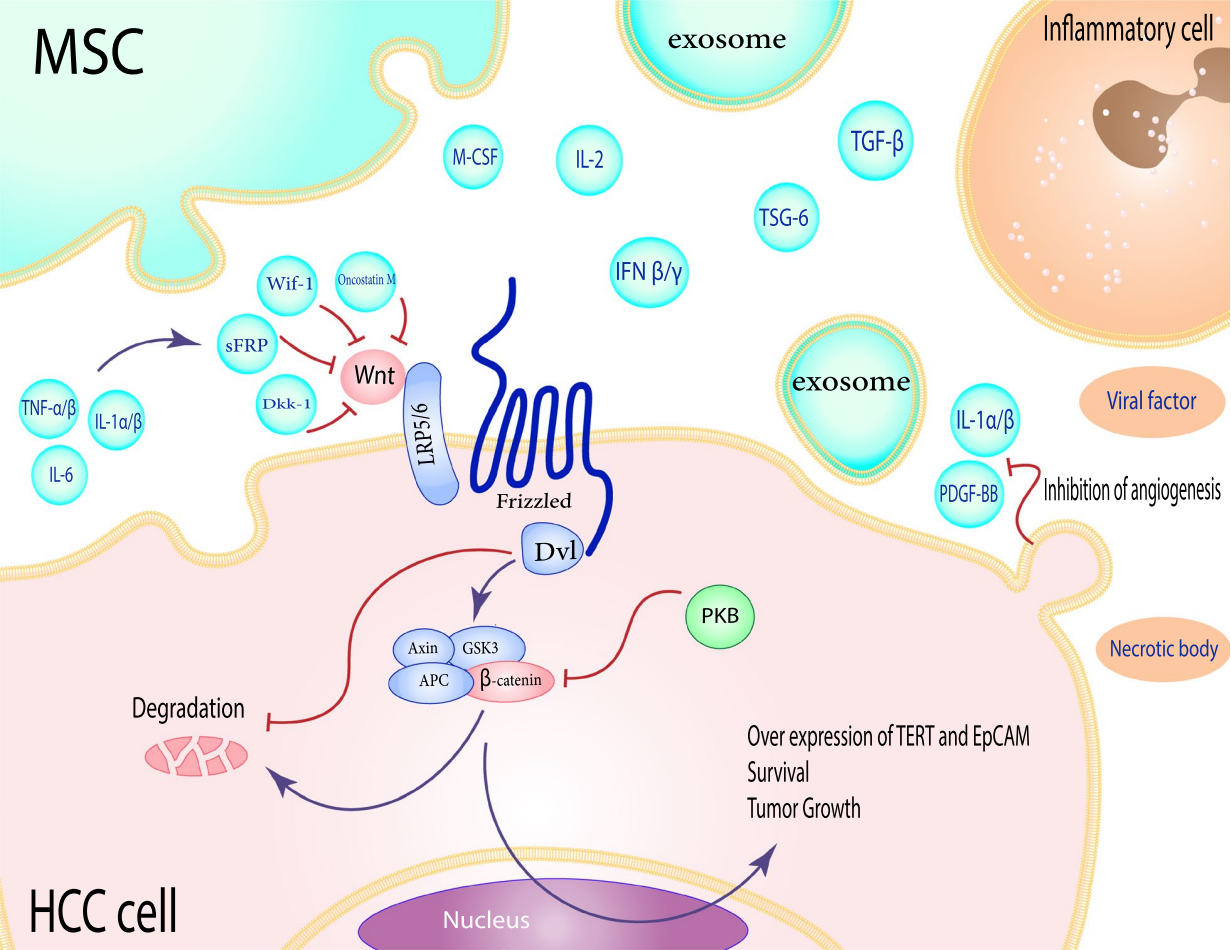
Schematic view of the Wnt signaling pathways between MSCs and HCC cells in inflammation microenvironment. Wnt is one of the signaling pathways that act in HCC for survival, tumor growth and overexpression of TERT and EpCAM. Wnt ligands activate this pathway by binding to the frizzled receptors and LRP5/6. Otherwise, Soluble Wnt antagonists including sFRP, Dkk-1, and Wif-1 modulated Initiation of Wnt signaling. MSCs had an inhibitory effect on the tumor cells proliferation through secreting these antagonists and TNF-α/β, IL-1α/β, and IL-6 for increasing of these inhibitory factors. Tumor cells can inhibit the production of PDGF-BB and IL-1β by MSCs, which in turn reduces angiogenesis

The canonical Wnt signaling pathway launches its promotion effects by stabilization of cytosolic β-catenin and its nuclear translocation as a transcriptional regulator, whereby it works as a co-activator of T-cell factor (TCF) proteins [20]. An initiating event is probably caused by the Wnt ligands, through binding to the Frizzled (Fzd) receptors and co-receptors, lipoprotein receptor-related protein (LRP) 5 and 6 [21]. Members of the Wnt proto-oncogene family can lead to tumor formation by accelerating proliferation and cell cycle progression [21]. Thus, for example, increased expression of the anti-apoptotic protein B-cell lymphoma 2 (BCL-2) and proliferating cell nuclear antigen (PCNA) has been linked to Wnt3a in cancer cells [22]. Soluble Wnt antagonists, including soluble frizzled-related protein (sFRP), dickkopf-related protein 1 (DKK-1) and Wnt inhibitory factor-1 (WIF-1) differentially modulate the initiation of Wnt signaling in both normal and malignant cell populations [23, 24] (Fig. 1).

Yet, in spite of many advances in signal transduction pathways, the impact of MSCs on Wnt signaling and their inhibitory effect on HCC cell growth remain largely unknown. To date, there is a lot of evidence that MSCs release some factors such as DKK-1 that compete with Wnt for binding to LRP5/6, thus inhibits the Wnt signaling pathway [11, 25].

Qiao et al. have elucidated the inhibitory properties of MSCs on the dynamic growth of human breast cancer cell lines. These results indicate that MSC-conditioned medium induces down-regulation of β-catenin, while overexpression of DKK-1 enhances the inhibitory effect of MSCs on the Wnt signaling pathway [25] (Fig. 1). Similarly, Zhu et al. reported the DKK-1 secretion by MSCs cause decreased proliferation of chronic myelogenous leukemia cancer cells [26]. Furthermore, mechanisms underlying the inhibition of proliferation and promotion of apoptosis have been investigated in human HepG2 cell lines. MSC-derived DKK-1 protein could inhibit the expression of Wnt-related factors, including c-Myc, BCL-2, survivin, and β-catenin, thus leads to the dysregulation of this signaling pathway [11].

Telomerase reverse transcriptase (TERT) gene is involved in regulating the stemness properties by influencing the telomere length maintenance of normal stem cells [27]. TERT expression is controlled through binding of β-catenin to its promoter region; thereby creating a regulatory link between telomerase function and Wnt signaling [28]. In addition, epithelial cell adhesion molecule (EpCAM) overexpression has been observed during liver development, regeneration and following the recovery from cirrhosis [29]. While absent in adult hepatocytes, this cell-surface tumor marker is identified as a direct transcriptional target of the Wnt signaling in HCC [30] (Fig. 1).

Abnormal cytoplasmic and nuclear accumulation of β-catenin, as well as the elevated level of Wnt receptor, frizzled-7 (FZD-7) in up to 90% of HCCs, revealed the importance of MSCs inhibitory properties on the Wnt signaling pathway [31, 32]. Hence, a great amount of effort has been put in this issue for approaching a stem cell-based therapy and also improving the effectiveness of inhibitory drugs in HCC patients [33].

### MSCs reduce inflammation in HCC

There is strong scientific evidence that infections with specific types of bacteria or viruses like hepatitis may increase the risk of HCC via induction of inflammation [34]. Similarly, contamination with aflatoxin B1 or environmental pollutants, including aromatic amines, vinyl chloride, polycyclic aromatic hydrocarbons, and nitrosamines are thought to be the general risk factors for HCC development [35].

The role of molecular changes in the acquisition of drug resistance phenotype may help to identify the prognostic markers and novel therapeutic approaches for HCC treatment [36]. The pro-tumorigenic effects of inflammation sustain via a feed-forward cytokine and chemokine network that attract more inflammatory cells toward the tumor microenvironment [37]. Multiple signal transduction pathways such as nuclear factor-kappa B (NF-κB) and c-Jun NH2-terminal kinase (JNK) are highly involved in the pathogenesis of viral diseases. For instance, the HCV core and HBV X (HBx) proteins are the most powerful inducers described to date, participating in the activation of NF-κB and activator protein 1 (AP-1) transcription factors [38]. Yen et al. have examined different signaling pathways related to the HBx-induced HCC development, as the overexpression of this protein lead to the upregulation of IκB kinase β (IKKβ), tuberous sclerosis complex 1 (TSC1) and mammalian target of rapamycin (mTOR) in HCC cells. Moreover, the elevated levels of pIKKβ, pTSC1, and pS6K1 are strongly correlated with a poor prognosis in HBV-associated hepatoma [39]. Here, we have tried to provide a comprehensive look into the role of toll-like receptors (TLRs), NF-κB and the JNK signaling pathways in HCC development.

#### TLR signaling in HCC

**Fig. 2:**
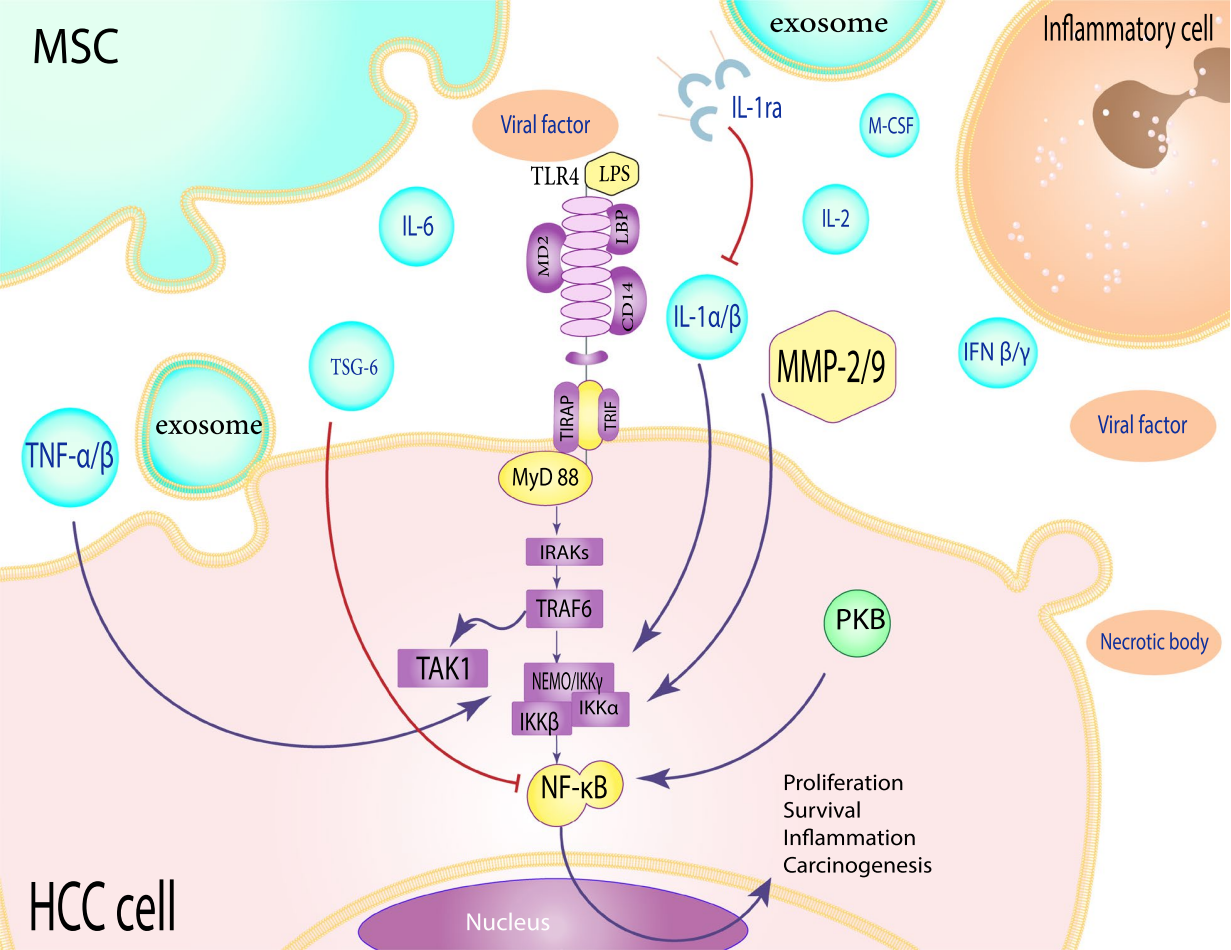
Schematic view of the TLR4 signaling pathways between MSCs and HCC cells in inflammation microenvironment. This signaling pathway activated by viral proteins and inflammatory conditions can ultimately lead to the activation of the NF-κB transcription factor. This transcription factor after entering the nucleus, induced expression of the factors involved in proliferation, survival inflammation, and carcinogenesis. The TSG-6 factor produced by MSCs leads to inhibition of this pathway, but IL-1α/β is an activator for NF-κB inducing. MSCs prevent the effect of IL-1α/β by the production of soluble receptors

TLRs are able to recognize the various patterns of pathogen- and host tissue–derived molecules and thus give rise to inflammation [40]. Their function has been mediated by the Toll/interleukin-1 receptor (TIR) domain [41]. TLR4 belonging to the pattern recognition receptor (PRR) superfamily, commonly play a central role in innate immunity. The 95 kDa integral membrane protein consists of an extracellular domain named leucine-rich repeat (LRR), as well as a cytoplasmic TIR domain, directing ligand recognition and signal transduction, respectively [42]. It has a limitless capacity for detecting pathogen-associated molecular patterns (PAMPs) or different classes of damage-associated molecular patterns (DAMPs), all lead to the production of required cytokines and pro-inflammatory proteins [43]. The adapter protein myeloid differentiation factor 88 (MyD88) acts as an intracellular signal transducer in cooperation with interleukin-1 receptor-associated kinase 1 and 4 (IRAK1/4), following the TLR4 activation [44]. Following the phosphorylation of these factors by TRAF6, TAK1 kinase is recruited into the cluster of signaling pathways [45]. Once activated, TAK1 activates the IκB kinase (IKK) complex that finally gives rise to NF-κB and JNK activation [46] (Fig. 2).

There are numerous factors that can activate the TLRs signaling in the liver, such as HBV, HCV, alcohol, and nonalcoholic steatohepatitis (NASH). Among TLRs, TLR4 and 9 suppress viral replication in HBV-transgenic mice [47, 48]. HBV replication cycle occurring via the upregulation of TLR2 thus results in a marked increase in tumor necrosis factor-alpha (TNF-α) production [49]. HCV core and NS3 proteins cause inflammation and activate TLR2 on immune cells to release cytokines [50, 51]. Moreover, excessive alcohol intake is highly associated with increased intestinal permeability and elevated levels of endotoxin [52]. In vivo studies have indicated that lipopolysaccharides (LPS) levels are significantly increased in cirrhosis, and can directly activate the hepatic stellate cells [53, 54]. LPS activates the TLR4-mediated pathway and thus, enhances the expression of proinflammatory cytokine [55, 56]. TLR4 and MyD88 deficient Mice have a considerable reduction in the number of chemically induced HCC, highlighting a direct influence of TLR signaling on hepatocarcinogenesis [57]. MyD88, as a TLR adapter protein, is necessary for NF-κB activation, and its downregulation is strongly associated with liver tumorigenesis suppression [58]. Therefore, the indispensable role of TLR4-MyD88 signaling in HCC development creates new approaches to disease prevention and treatment [36] (Fig. 2).

#### NF-κB signaling activation in HCC

NF-κB is present broadly in the majority of cells and is a key transcription factor in cellular proliferation, differentiation, carcinogenesis, and apoptosis [59]. Across several studies, researchers have shown that NF-κB contributes to tumorigenesis by affecting the tumor cells and tumor-associated inflammatory cells mechanisms [60–62]. Overall, the inhibition of NF-κB activity provides convincing evidence of a novel therapeutic implication for HCC [63]. Numerous proinflammatory stimuli upregulate NF-κB, by phosphorylation of IKK and degradation of the κB inhibitor (IκB) proteins [64]. Members of the TNF family, like the B-cell activating factor (BAFF), CD40 ligand, and lymphotoxin β (LTβ) demonstrating a key role in activating the NF-κB pathway [65].

Active NF-κB controls the expression of several anti-apoptotic proteins, including cIAPs, c-FLIP, and BCL-X that are essential for maintaining cancer cells [66]. Tumor cells with continuous NF-κB activity are highly resistant to antitumor agents and thus, specific inhibition of NF-κB may promote cell sensitivity to applied treatment [67]. Overexpression of IKKα and IKKβ kinases, essential for NF-κB activation, is definitely needed for the acquisition of malignant HCC properties [68]. Surprisingly, downregulation of NF-κB and p-IκBα has been demonstrated in treated HCC cell lines, but how MSCs can regulate the NF-κB signaling pathway mostly remain unclear [69]. However, the role of various factors present in the MSCs-conditioned media should not be ignored for the downregulation of the NF-κB pathway in tumor cells (Fig. 2).

#### JNK Signaling activation in HCC

**Fig. 3:**
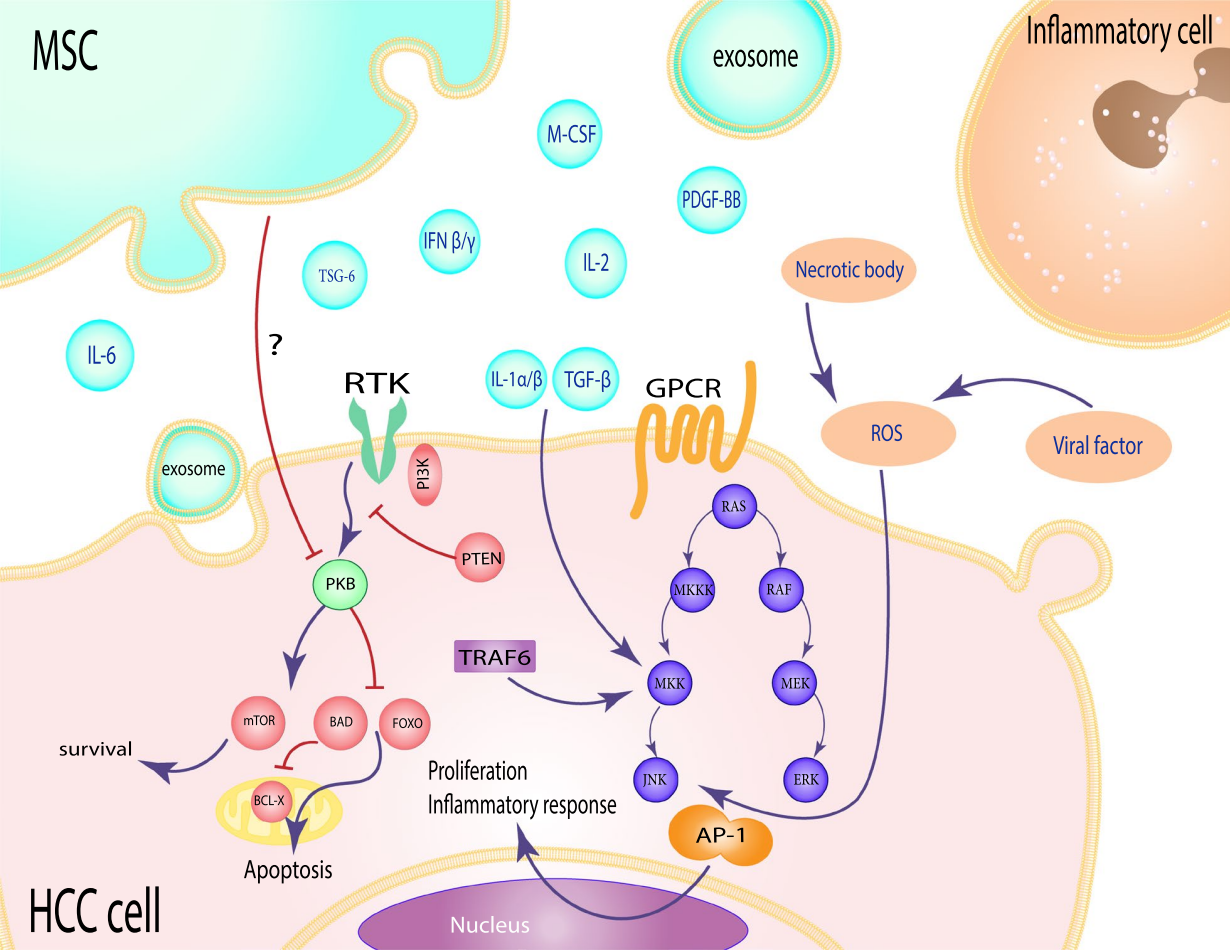
Schematic view of the AP-1 and PKB signaling pathways between MSCs and HCC cells in inflammation microenvironment. The pathway AP-1 transcription factor activates through various factors, including viral proteins, necrotic factors that produce ROS, IL-1α/β and TGF-β produced from MSCs, and TRAF6 is activated from the TLR inflammatory pathway. MSCs inhibit PKB pathogenic pathway so that the tumor cell goes to apoptosis

The JNK is a major member of the mitogen-activated kinases (MAPKs) family, together with extracellular regulated kinase (ERK) and p38 [70]. JNKs activation in response to stress signals and pro-inflammatory stimuli would eventually enhance the phosphorylation of MAPK kinases MKK4 and MKK7 [71]. They also phosphorylate the transcription factors c-Jun, JunD, as well as the activating transcription factor (ATF), which contribute to AP-1 complex formation [72]. A number of studies have emphasized the importance of active JNK signaling for hepatocyte proliferation and apoptosis [73]. It is clearly recognized that cellular immortality occurs due to a DNA mutation in association with JNK activation [74]. Phosphorylation of c-Jun proto-oncogene via the JNK pathway may promote the development and progression of HCC disease [75] (Fig. 3). Approximately 70% of HCC tissues show positive immunostaining for phosphorylated JNK, which revealed the vital role of this protein kinase in human HCC pathogenesis [36].

Tong et al. described the spontaneous occurrence of intestinal tumors in JNK1 knockout mice, suggesting a suppressor role for JNK1 in intestinal tumor development [76]. Possibly, signal transmission by a number of growth factors provokes JNKs by activating the receptor tyrosine kinases, as well as the phosphorylation of various receptors [77]. In vitro modeling of HCC studies revealed the pathogenic impact of several HBV and HCV proteins on JNK activation [78]. Viral proteins such as the HBV X and HCV core proteins generally induce the accumulation of reactive oxygen species (ROS) in hepatocytes [79, 80]. Sustained production of ROS then activates the JNK via either stimulating upstream MEK kinases (MEKKs) or inhibiting MAP kinase phosphatases (MKPs) [81, 82]. Thus, ROS-JNK signaling is found to be a major determinant of HCC progression [83, 84] (Fig. 3).

Molecules involved in MSCs homing to tumor microenvironments are mainly consisting of several inflammatory cytokines (TNF-α, IL-1β, IFN-γ, and IL-6), chemokines (CXCR4, CXCL7, CXCL6, and CXCL5) and growth factors (PDGF, HGF), [85, 86]. Interestingly, MSCs are able to suppress tumor cell growth by releasing inhibitory factors-containing exosomes against the proliferation of signaling pathways [87]. Moreover, the secretion of IL-1ra by MSCs prevents the IL-α / β activity by producing TSG-6, followed by the downregulation of NF-κB signaling and decreased the production of inflammatory cytokines. Secretion of prostaglandin E2 (PGE2) is another effective way of reducing inflammation by MSCs that accomplished by the production of IL-10, as a potent anti-inflammatory cytokine [88, 89]. Khakoo et al. indicated that MSCs inhibit the target cell PKB signaling activity through a contact-dependent way [16]. In total, the downregulation of NF-κB is suggested to be a beneficial behavior of MSCs to inhibit tumor cell proliferation and invasion (Fig. 2).

### Hippo signaling pathway in HCC

The control of organ size following multiple cell proliferation is fundamental through the developmental process, as an increased organ size due to injury should be returned to normal values by regeneration [90]. The Hippo signaling pathway is mainly involved in the regulation of organ growth and overall organ size. This signal transduction cascade mediates cell proliferation, differentiation, and survival, as well as the homeostasis and development in cooperation with other signaling interactions. Any disruption in key components of this pathway has a great potential in tumor formation and in particular HCC [91]. Recent studies have shown the essential role of Hippo signaling in stem cell regulation, especially in liver cancer progenitor cells that ultimately lead to hepatocarcinogenesis [92, 93].

This signaling pathway consists of a number of highly conserved components: the protein kinase Hippo (Hpo), the scaffold protein Salvador (Sav), the protein kinase Warts (Wts), and Mob as a tumor suppressor (Mats) that are homologous to mammalian Mst1/2, WW45, LATS1/2, and Mob1, respectively. Their essential role in organ size control is considered as the main reason for possessing an evolutionary conservation pattern, as any mutation in genes encoding Hippo signaling proteins can lead to significant changes in organ morphology or growth parameters [94].

Similar to other pathways, Hippo signaling is negatively controlled by phosphorylation via the growth modulator Yorkie (Yki) or its mammalian homolog Yes-associated protein (YAP). So that the overexpression of this factor or mutations in core Hippo pathway components resulted in the activation of two major cell proliferation markers, Cyclin E and Drosophila inhibitor of apoptosis protein 1 (DIAP1) to prevent cell death [95, 96]. In recent studies, YAP has been reported as an oncogene in many human tumors, especially for those with HCC. Zhao et al. reported a significant increase in YAP expression in nearly 54% of patients with HCC compared to normal liver tissue. This study concluded that the uncontrolled YAP expression could play an increasingly important role through HCC development [97]. The same results were also observed in the study by Xu et al., in which 62% of examined samples showed a higher level of protein expression, associated with tumor aggressiveness and unfavorable prognosis of HCC [98].

Since the Hippo signaling pathway has recently been discovered, more extensive studies should be performed related to the effect of MSCs on HCC development. A novel study suggested that the transcriptional co-activator with PDZ-binding motif (TAZ), one of the nuclear effectors of this signaling cascade, can effectively modulate cell proliferation and the formation of epithelial–mesenchymal transition (EMT) in HCC [99]. The expression of TAZ is significantly increased in HCC, as the knockdown of this downstream regulatory target can prevent tumorigenesis and reduces HCC cell migration. The EMT process occurs as a result of decreased expression of epithelial genes such as E‐cadherin and the increased expression of mesenchymal genes such as N‐cadherin, vimentin, Snail, and Slug [100]. Eventually, the two most important factors of the Hippo signaling pathway, TAZ, and YAP, have a significant impact on the key step of transdifferentiation process development [101]. Surprisingly, the hypoxic state of tumor microenvironment cause increased secretion of prostaglandin E2 (PGE2) from the surrounded MSCs and ultimately increase the YAP protein level in HCC cells [102, 103]. Due to a lack of research that comprehensively analyze the effect of MSCs on Hippo signaling, genetic modification of mesenchymal cells or considering these multipotent stromal cells as drug delivery vehicles may possibly decrease the negative feedback effects of this signaling cascade and prevent HCC tumor progression.

## Discussion

HCC is considered as a common malignant condition, caused by impaired hepatic stem cell function [104]. There are multiple signal transduction pathways, regulate the self-renewal, differentiation and cell fate specifications of precursor cells. Therefore, understanding the exact mechanism of these signaling cascades may provide an alternative way to explore the link between stemness and tumorigenesis [105]. Increasing evidence has indicated that adult stem cells may be considered as an effective therapeutic tool for cancer therapy [106]. Certain studies have described the intrinsic ability of MSCs to decrease the growth rate of numerous cancer cell types [107]. Ma et al. directly injected human umbilical cord MSCs (hUC-MSCs) xenograft transplanted into immunodeficient mice, bearing MDA-MB-231 breast cancer stem cells. Surprisingly, the hUC-MSCs lead to reduced tumor volume and tumor weight in mice models [108]. Zhang et al. investigation on hUC-MSCs, transfected with IL-21 gene determined the ability of such modified cells to suppress the proliferation of ovarian cancer cells in vitro and in vivo [109]. Subramanian et al. research confirmed that hUC-MSCs did not transform into tumor-associated fibroblasts, making them invulnerable than bone marrow MSCs [110].

MSCs are thought to suppress tumor growth in different ways: induction of inflammatory cell infiltration, angiogenesis inhibition, suppression of Wnt, NF-κB, and PKB signaling molecules. Paracrine effects of these multipotent cells also lead to cell cycle arrest and apoptosis via Dkk1 and oncostatin M activity [11, 25, 69, 111–114]. Ryu et al. reported that adipose tissue-derived MSCs are able to grow at high cell density, followed by the prevention of MCF-7 cell growth through IFN-β production [115]. Moreover, MSCs primed with IFN-γ or cultured with tri-dimensional systems can express TNF-related apoptosis-inducing ligand (TRAIL), which triggers tumor cell-specific apoptosis [116, 117]. The result of Loebinger et al. study confirmed that MSCs could reduce colony formation of squamous and adenocarcinoma lung cancer cells by expressing TRAIL. This protein binds to its receptors and leading to the activation apoptosis pathway in tumor cells. [118].

In addition, Zhao et al. study indicated that 3D cultured MSCs have an inhibitory effect on HepG2 cell proliferation, by releasing the IL-24 and the activation of the JAK1-STAT3 signaling pathway. This study suggested a more feasible way for MSCs to inhibit tumor cell growth and invasion [119].

In contrast to numerous research that expresses the inhibitory role of MSCs on tumor cells, some reports showed enhanced tumor growth due to the homing capability of MSCs to tumor sites [120]. The Supportive of confounding behavior effect of MSCs toward tumor cells are highly associated with the dose of injected multipotent stromal cells to targeted organs [113]. Depending on the exact experimental conditions, MSCs can exert tumor-promoting or tumor-limiting outcomes (Table 1). Various hypotheses have been recommended to explain the dualistic behavior of MSCs in cancer. TLR4-primed MSCs are polarized into pro-inflammatory MSC1 phenotype, whereas TLR3-primed MSCs are specialized as classical immunosuppressive MSC2 [121]. In cancer models, MSC1-based treatment of established tumors in a case of strong immune system attenuates tumor growth and metastasis, while MSC2-treated animals would display an increase in tumor growth rate and metastatic potential [122]. As both hypotheses are not mutually exclusive likely both are concomitantly true, making our prediction about the role of MSCs on the cancerous process extremely difficult. However, the reasons behind this apparent discrepancy need to be further investigated.

**Table 1:** Dual effect of MSCs on HCC

References	Effects	Cell lines	Results
Hou et al. [11]	Inhibit	HEK 293 and HepG2 cell lines	Wnt signaling pathway may have a function in MSC-mediated tumor cell inhibition
Yan-rong Lu et al. [113]	Inhibit	murine hepatoma H22, lymphoma (YAC-1 and EL-4) and rat insulinoma INS-1 cell lines	MSCs had potential inhibitory effects on tumor cell growth in vitro and in vivo without host immunosuppression, by inducing apoptotic cell death and G0/G1 phase arrest of cancer cells
Li et al. [132]	Inhibit	MHCC97-H cell line	MSC could be useful in controlling metastatic recurrence of HCC
Zhao et al. [133]	Inhibit	HepG2, Huh7, SMMC7721, and Bel7402 cell lines	inhibited HCC cell proliferation and division induced HCC cell death through the downregulation of Akt signaling
El Asmar et al. [105]	Inhibit	DENA and CCl4 induction in rat model	tumor suppressive effects as evidenced by down regulation of Wnt signaling target genes concerned with anti-apoptosis, mitogenesis, cell proliferation and cell cycle regulation
Li et al. [134]	Inhibit	MHCC97-H cell line	The metastatic potential of tumor cells was downregulated after hMSC engraftment and hMSCs induce further tumor cells apoptosis
Qiao et al. [135]	Inhibit	H7402 and HepG2 cell lines	The Wnt signaling pathway may have a role in hMSC-mediated targeting and tumor cell inhibition
Qiao et al. [69]	Inhibit	H7402 cell line	NF‐κB downregulation is one of reasons for the depression of tumor cell proliferation mediated by hMSCs
Bruno et al. [136]	Inhibit	HepG2 cell line	MVs from human MSCs inhibited in vitro cell growth
Ma et al. [137]	Inhibit	H22 cell line	BMSCs pulsed with TEX could enhance its antitumor activities
Abd-Allah et al. [138]	Inhibit	HEPA 1-6 cell line	MSCs could inhibit cell division of HCC and potentiate their death
Seyhoun et al. [139]	Inhibit	HepG2 cell line	Combination therapy MSCs and sorafenib to the treatment of HCC that significantly improves the results
Hajighasemlou et al. [140]	Inhibit	HepG2 cell line	Local injection of MSCs can be used as cell therapy to fight neoplasms
Hernanda et al. [141]	Promote	Huh7 cell line	MSCs are enriched in human HCC tumor compartment and could exert trophic effects on tumor cells
Yan et al. [142]	Promote	MHCC97L cell line	HCC progression, and may be a potential therapeutic target
Gong et al. [143]	Promote	HepG-2 cell line	promote the growth of microvascular in hepatoma cells
Jing et al. [144]	Promote	SMMC-7721 cell line	MSCs in tumor inflammatory microenvironment could promote tumor metastasis through TGFβ-induced EMT
Bhattacharya et al. [145]	Promote	SK-Hep1 cell line	stimulation of cancer-associated fibroblasts and EMT markers
Liu et al. [146]	Promote	HCCLM3 cell line	significantly enhance the tumor cell metastasis, which was due to the EMT of HCC cells induced by TGF-β

*CCl4* carbon tetrachloride, *DENA* diethylenetriamine, *EMT* epithelial to mesenchymal transition, *HCC* Hepatocellular carcinoma, *hMSCs* human mesenchymal stem cells, *MVs* Microvesicles, *TGF-β* transforming growth factor beta

On the other hand, in some instances, tumor cells can inhibit the PDGF-BB and IL-1β production by MSCs, which in turn reduces the angiogenesis and tumor growth [123] (Fig. 1). In a recent study by Pan et al., trophic factors released from MSCs suppress the translation initiation factor eIF4E via the MAPK signaling pathway. Therefore, the secretion of vascular endothelial growth factor (VEGF) could be a revolutionary new way of treating cancer by altering the tumor cell fate specifications [124]. MSCs also produce the exosomes-loaded with miR-122 that significantly increases the sensitivity of HCC cells to sorafenib, leading to *in vivo* tumor growth arrest [125].

Targeted localization of MSCs in tumor sites will have a significant impact on the achievement of specific antitumor therapy [126]. MSCs exhibit an intrinsic homing property, enabling a collective cell migration to inflammatory sites. The exploitation of this process will be a valuable asset to directed therapy [127]. The capability to express exogenous gene products, genetic stability and allogeneic properties turn MSCs into efficient carriers for antitumor therapy [128]; previously demonstrated not only in tumor models but also in a wide range of other diseases such as graft-versus-host disease, multiple sclerosis, and arthritis [129–131].

Therefore, MSCs have multiple immunosuppressant properties that required for tumor growth inhibition and also likely to be effective in cancer treatment via producing several factors such as microRNAs. Nevertheless, more detailed information about the interactions between MSCs and tumor cells will help us to develop novel therapeutic approaches in the future. Yet, an important issue remains unanswered regarding the time and the approximate number of such regulatory cells that are delivered to target organs. However, their role as an adjunct in patients with liver tumors looks hopeful and promising.

## Conclusions

Recent studies have suggested the use of cell-based therapeutic approaches for cancer treatment. Here we discussed the inhibitory role of normal human MSCs on HepG2 cell proliferation, proposing the valuable impact of these multipotent stromal cells on liver cancer therapy. While the exact molecular mechanisms between the MSCs and tumors cells are still unknown, but the overall results of several studies revealed the suppression effect of MSCs on HCC through both inflammation mediators and vital signaling pathways. Therefore, further research needed to develop a novel clinical application of MSCs for HCC patients.

## Data Availability

The primary data for this study is available from the authors on direct request.

## Abbreviations

AP-1: activator protein-1
APC: adenomatous polyposis coli
CD14: cluster of differentiation 14
BAD: Bcl-2-associated death promoter
DKK-1: dickkopf 1
Dvl: dishevelled
EpCAM: epithelial cell adhesion molecule
ERK: extracellular signal-regulated kinases
FOXO: forkhead box
GPCR: G protein-coupled receptors
GSK3: glycogen synthase kinase 3
IKK: I-kappa-B kinase
IRAKs: IL-1 receptor-associated kinases
IL: interleukin
IFN: interferon
JNK: c-Jun N-terminal kinases
LBP: lipopolysaccharide binding protein
LRP5/6: low density lipoprotein receptor-related protein 5/6
MD2: myeloid differentiation factor 2
MyD88: myeloid differentiation primary response gene 88
mTOR: mammalian target of rapamycin
M-CSF: macrophage-colony stimulating factor
MMP: matrix metalloproteinases
MEK: MAPK/ERK kinase
MKKK: mitogen-activated protein kinase kinase kinase
MKK: mitogen-activated protein kinase kinase
NF-κB: nuclear factor
NEMO: NF-kappa-B essential modulator
PI3K: phosphoinositide 3-kinase
PTEN: phosphatase and tensin homolog
PKB: protein kinase B
PDGF: platelet-derived growth factor
RTK: receptor tyrosine kinases
sFRP: soluble frizzled related proteins (sFRP)
TERT: telomerase reverse transcriptase
TRIF: TIR-domain-containing adapter-inducing interferon-β
TNFa: tumor necrosis factor a
TLR4: toll-like receptor 4
TIRAP: TIR domain-containing adaptor protein
TRAF6: TNF receptor associated factor 6
TGFβ: transforming growth factor beta
TAK1: TGFβ- activated kinase
TSG-6: TNF-stimulated gene 6
Wif-1: Wnt inhibitory factor-1

## References

[1] Ayuso C, Rimola J, Vilana R, Burrel M, Darnell A, García-Criado Á (2018). Diagnosis and staging of hepatocellular carcinoma (HCC): current guidelines. Eur J Radiol.

[2] El-Serag HB (2012). Epidemiology of viral hepatitis and hepatocellular carcinoma. Gastroenterology.

[3] Janevska D, Chaloska-Ivanova V, Janevski VJ (2015). Hepatocellular carcinoma: risk factors, diagnosis and treatment. Open Access Maced J Med Sci.

[4] Serhal R, Saliba N, Hilal G, Moussa M, Hassan GS, El Atat O (2019). Effect of adipose-derived mesenchymal stem cells on hepatocellular carcinoma: in vitro inhibition of carcinogenesis. World J Gastroenterol.

[5] Crissien AM, Frenette CJG (2014). Current management of hepatocellular carcinoma. Hepatology.

[6] El-Ansary M, Abdel-Aziz I, Mogawer S, Abdel-Hamid S, Hammam O, Teaema S (2012). Phase II trial: undifferentiated versus differentiated autologous mesenchymal stem cells transplantation in Egyptian patients with HCV induced liver cirrhosis. Stem Cell Rev Rep.

[7] Moradian Tehrani R, Verdi J, Noureddini M, Salehi R, Salarinia R, Mosalaei M (2018). Mesenchymal stem cells: a new platform for targeting suicide genes in cancer. J Cell Physiol.

[8] Elahi KC, Klein G, Avci-Adali M, Sievert KD, MacNeil S, Aicher WK (2016). Human mesenchymal stromal cells from different sources diverge in their expression of cell surface proteins and display distinct differentiation patterns. Stem cells Int.

[9] Ridge SM, Sullivan FJ, Glynn SA (2017). Mesenchymal stem cells: key players in cancer progression. Mol Cancer.

[10] Cai C, Hou L, Zhang J, Zhao D, Wang Z, Hu H (2017). The inhibitory effect of mesenchymal stem cells with rAd-NK4 on liver cancer. Appl Biochem Biotechnol.

[11] Hou L, Wang X, Zhou Y, Ma H, Wang Z, He J (2014). Inhibitory effect and mechanism of mesenchymal stem cells on liver cancer cells. Tumor Biol.

[12] Martin J, Dufour J-F (2008). Tumor suppressor and hepatocellular carcinoma. World J Gastroenterol.

[13] Ho C, Wang C, Mattu S, Destefanis G, Ladu S, Delogu S (2012). AKT and N-Ras co-activation in the mouse liver promotes rapid carcinogenesis via mTORC1, FOXM1/SKP2, and c-Myc pathways. Hepatology (Baltimore, MD).

[14] Vilchez V, Turcios L, Marti F, Gedaly R (2016). Targeting Wnt/β-catenin pathway in hepatocellular carcinoma treatment. World J Gastroenterol.

[15] Galuppo R, Maynard E, Shah M, Daily MF, Chen C, Spear BT (2014). Synergistic inhibition of HCC and liver cancer stem cell proliferation by targeting RAS/RAF/MAPK and WNT/β-catenin pathways. Anticancer Res.

[16] Khakoo AY, Pati S, Anderson SA, Reid W, Elshal MF, Rovira II (2006). Human mesenchymal stem cells exert potent antitumorigenic effects in a model of Kaposi’s sarcoma. J Exp Med.

[17] Hajighasemlou S, Pakzad S, Ai J, Muhammadnejad S, Mirmoghtadaei M, Hosseinzadeh F (2018). Characterization and validation of hepatocellular carcinoma (HCC) xenograft tumor as a suitable liver cancer model for preclinical mesenchymal stem cell studies. Asian Pac J Cancer Prev.

[18] Dhanasekaran R, Bandoh S, Roberts LR (2016). Molecular pathogenesis of hepatocellular carcinoma and impact of therapeutic advances. F1000Research.

[19] Wands JR, Kim M (2014). WNT/β-catenin signaling and hepatocellular carcinoma. Hepatology.

[20] Shang S, Hua F, Hu Z-W (2017). The regulation of β-catenin activity and function in cancer: therapeutic opportunities. Oncotarget..

[21] MacDonald BT, He X (2012). Frizzled and LRP5/6 receptors for Wnt/β-catenin signaling. Cold Spring Harbor perspectives in biology..

[22] Muncan V, Sansom OJ, Tertoolen L, Phesse TJ, Begthel H, Sancho E (2006). Rapid loss of intestinal crypts upon conditional deletion of the Wnt/Tcf-4 target gene c-Myc. Mol Cell Biol.

[23] Byun T, Karimi M, Marsh J, Milovanovic T, Lin F, Holcombe R (2005). Expression of secreted Wnt antagonists in gastrointestinal tissues: potential role in stem cell homeostasis. J Clin Pathol.

[24] Ramachandran I, Thavathiru E, Ramalingam S, Natarajan G, Mills W, Benbrook D (2012). Wnt inhibitory factor 1 induces apoptosis and inhibits cervical cancer growth, invasion and angiogenesis in vivo. Oncogene.

[25] Qiao L, Xu Z, Zhao T, Ye L, Zhang X (2008). Dkk-1 secreted by mesenchymal stem cells inhibits growth of breast cancer cells via depression of Wnt signalling. Cancer Lett.

[26] Zhu Y, Sun Z, Han Q, Liao L, Wang J, Bian C (2009). Human mesenchymal stem cells inhibit cancer cell proliferation by secreting DKK-1. Leukemia.

[27] Flores I, Blasco MA (2010). The role of telomeres and telomerase in stem cell aging. FEBS Lett.

[28] Ozturk MB, Li Y, Tergaonkar V (2017). Current insights to regulation and role of telomerase in human diseases. Antioxidants..

[29] Yamashita T, Ji J, Budhu A, Forgues M, Yang W, Wang HY (2009). EpCAM-positive hepatocellular carcinoma cells are tumor-initiating cells with stem/progenitor cell features. Gastroenterology.

[30] Yamashita T, Budhu A, Forgues M, Wang XW (2007). Activation of hepatic stem cell marker EpCAM by Wnt–β-catenin signaling in hepatocellular carcinoma. Cancer Res.

[31] Merle P, de la Monte S, Kim M, Herrmann M, Tanaka S, Von Dem Bussche A (2004). Functional consequences of frizzled-7 receptor overexpression in human hepatocellular carcinoma. Gastroenterology.

[32] Inagawa S, Itabashi M, Adachi S, Kawamoto T, Hori M, Shimazaki J (2002). Expression and prognostic roles of β-catenin in hepatocellular carcinoma: correlation with tumor progression and postoperative survival. Clin Cancer Res.

[33] Dahmani R, Just P-A, Perret C (2011). The Wnt/β-catenin pathway as a therapeutic target in human hepatocellular carcinoma. Clin Res Hepatol Gastroenterol.

[34] Hoshida Y, Fuchs BC, Bardeesy N, Baumert TF, Chung RT (2014). Pathogenesis and prevention of hepatitis C virus-induced hepatocellular carcinoma. J Hepatol.

[35] Rapisarda V, Loreto C, Malaguarnera M, Ardiri A, Proiti M, Rigano G (2016). Hepatocellular carcinoma and the risk of occupational exposure. World J Hepatol.

[36] Maeda S (2010). NF-κB, JNK, and TLR signaling pathways in hepatocarcinogenesis. Gastroenterol Res Pract.

[37] Grivennikov SI, Greten FR, Karin M (2010). Immunity, inflammation, and cancer. Cell.

[38] Sun B, Karin M (2008). NF-κB signaling, liver disease and hepatoprotective agents. Oncogene.

[39] Yen C-J, Lin Y-J, Yen C-S, Tsai H-W, Tsai T-F, Chang K-Y (2012). Hepatitis B virus X protein upregulates mTOR signaling through IKKβ to increase cell proliferation and VEGF production in hepatocellular carcinoma. PLoS ONE.

[40] Ippagunta SK, Pollock JA, Sharma N, Lin W, Chen T, Tawaratsumida K (2018). Identification of Toll-like receptor signaling inhibitors based on selective activation of hierarchically acting signaling proteins. Sci Signal..

[41] Narayanan KB, Park HH (2015). Toll/interleukin-1 receptor (TIR) domain-mediated cellular signaling pathways. Apoptosis.

[42] Botos I, Segal DM, Davies DRJS (2011). The structural biology of Toll-like receptors. J Anim Sci Technol.

[43] Shabani F, Farasat A, Mahdavi M, Gheibi N (2018). Calprotectin (S100A8/S100A9): a key protein between inflammation and cancer. Inflamm Res.

[44] Vollmer S, Strickson S, Zhang T, Gray N, Lee K, Rao V (2017). The mechanism of activation of IRAK1 and IRAK4 by interleukin-1 and Toll-like receptor agonists. Biochem J.

[45] Dunne A, Carpenter S, Brikos C, Gray P, Strelow A, Wesche H (2010). IRAK1 and IRAK4 promote phosphorylation, ubiquitination and degradation of MyD88 adapter-like (MAL). J Biol Chem.

[46] Israël A (2010). The IKK complex, a central regulator of NF-κB activation. Cold Spring Harbor Persp Biol.

[47] Suslov A, Boldanova T, Wang X, Wieland S, Heim MH (2018). Hepatitis B virus does not interfere with innate immune responses in the human liver. Gastroenterology.

[48] Isogawa M, Robek MD, Furuichi Y, Chisari FV (2005). Toll-like receptor signaling inhibits hepatitis B virus replication in vivo. J Virol.

[49] Visvanathan K, Skinner NA, Thompson AJ, Riordan SM, Sozzi V, Edwards R (2007). Regulation of Toll-like receptor-2 expression in chronic hepatitis B by the precore protein. Hepatology.

[50] Modhiran N, Watterson D, Muller DA, Panetta AK, Sester DP, Liu L (2015). Dengue virus NS1 protein activates cells via Toll-like receptor 4 and disrupts endothelial cell monolayer integrity. Sci Transl Med.

[51] Dolganiuc A, Oak S, Kodys K, Golenbock DT, Finberg RW, Kurt-Jones E (2004). Hepatitis C core and nonstructural 3 proteins trigger toll-like receptor 2-mediated pathways and inflammatory activation. Gastroenterology.

[52] Seki E, Schnabl B (2012). Role of innate immunity and the microbiota in liver fibrosis: crosstalk between the liver and gut. J Physiol.

[53] Seki E, De Minicis S, Österreicher CH, Kluwe J, Osawa Y, Brenner DA (2007). TLR4 enhances TGF-β signaling and hepatic fibrosis. Nat Med.

[54] Douhara A, Moriya K, Yoshiji H, Noguchi R, Namisaki T, Kitade M (2015). Reduction of endotoxin attenuates liver fibrosis through suppression of hepatic stellate cell activation and remission of intestinal permeability in a rat non-alcoholic steatohepatitis model. Mol Med Rep.

[55] Zhu T, Wenjuan T, Tan Z, Liu L (2015). Effects of inhibited expression of IRF3 in LPS-stimulated Kupffer cells on the activation of signal transduction pathways. Chin J Microbiol Immunol.

[56] Yu L-X, Ling Y, Wang H-Y (2018). Role of nonresolving inflammation in hepatocellular carcinoma development and progression. NPJ Prec Oncol.

[57] Seki E, Brenner DA (2008). Toll-like receptors and adaptor molecules in liver disease: update. Hepatology.

[58] Trucco LD, Roselli E, Araya P, Nuñez NG, Mena HA, Bocco JL (2017). Downregulation of adaptor protein MyD88 compromises the angiogenic potential of B16 murine melanoma. PLoS ONE.

[59] Chen F, Castranova V, Shi X (2001). New insights into the role of nuclear factor-κB in cell growth regulation. Am J Pathol.

[60] Stein I, Bramovitch R, Amit S, Kasem S, Gutkovich-Pyest E, Urieli-Shoval S (2004). NF-kappaB functions as a tumour promoter in inflammation-associated cancer. Nature.

[61] Luo J-L, Kamata H, Karin M (2005). IKK/NF-κB signaling: balancing life and death—a new approach to cancer therapy. J Clin Investig.

[62] Greten FR, Arkan MC, Bollrath J, Hsu L-C, Goode J, Miething C (2007). NF-κB is a negative regulator of IL-1β secretion as revealed by genetic and pharmacological inhibition of IKKβ. Cell.

[63] Prabhu L, Mundade R, Korc M, Loehrer PJ, Lu T (2014). Critical role of NF-κB in pancreatic cancer. Oncotarget..

[64] Gupta SC, Sundaram C, Reuter S, Aggarwal BB (2010). Inhibiting NF-κB activation by small molecules as a therapeutic strategy. BBA Gene Regul Mech.

[65] Gardam S, Brink R (2014). Non-canonical NF-κB signaling initiated by BAFF influences B cell biology at multiple junctures. Front immunol.

[66] Ranjan K, Pathak C (2016). FADD regulates NF-κB activation and promotes ubiquitination of cFLIP L to induce apoptosis. Sci Rep.

[67] Baud V, Karin M (2009). Is NF-κB a good target for cancer therapy? Hopes and pitfalls. Nat Rev Drug Discov.

[68] Jiang R, Xia Y, Li J, Deng L, Zhao L, Shi J (2010). High expression levels of IKKα and IKKβ are necessary for the malignant properties of liver cancer. Int J Cancer.

[69] Qiao L, Zhao TJ, Wang FZ, Shan CL, Ye LH, Zhang XD (2008). NF-κB downregulation may be involved the depression of tumor cell proliferation mediated by human mesenchymal stem cells 1. Acta Pharmacol Sin.

[70] Munshi A, Ramesh R (2013). Mitogen-activated protein kinases and their role in radiation response. Genes Cancer.

[71] Eaton GJ (2015). Apoptosis signal-regulating kinase 1 modulates endochondral bone formation and osteoarthritis progression.

[72] Shaulian E, Karin M (2002). AP-1 as a regulator of cell life and death. Nat Cell Biol.

[73] Seki E, Brenner DA, Karin M (2012). A liver full of JNK: signaling in regulation of cell function and disease pathogenesis, and clinical approaches. Gastroenterology.

[74] Maeda S, Omata M (2008). Inflammation and cancer: role of nuclear factor-kappaB activation. Cancer Sci.

[75] Trierweiler C, Hockenjos B, Zatloukal K, Thimme R, Blum H, Wagner E (2016). The transcription factor c-JUN/AP-1 promotes HBV-related liver tumorigenesis in mice. Cell Death Differ.

[76] Tong C, Yin Z, Song Z, Dockendorff A, Huang C, Mariadason J (2007). c-Jun NH2-terminal kinase 1 plays a critical role in intestinal homeostasis and tumor suppression. Am J Pathol.

[77] Katz M, Amit I, Yarden Y (2007). Regulation of MAPKs by growth factors and receptor tyrosine kinases. BBA Mol Cell Res.

[78] Choi J, Corder NL, Koduru B, Wang Y (2014). Oxidative stress and hepatic Nox proteins in chronic hepatitis C and hepatocellular carcinoma. Free Radical Biol Med.

[79] Tarocchi M, Polvani S, Marroncini G, Galli A (2014). Molecular mechanism of hepatitis B virus-induced hepatocarcinogenesis. WJG.

[80] Suhail M, Abdel-Hafiz H, Ali A, Fatima K, Damanhouri GA, Azhar E (2014). Potential mechanisms of hepatitis B virus induced liver injury. WJG.

[81] Chen F, Beezhold K, Castranova V (2009). JNK1, a potential therapeutic target for hepatocellular carcinoma. BBA Rev Cancer.

[82] Neuveut C, Wei Y, Buendia MA (2010). Mechanisms of HBV-related hepatocarcinogenesis. J Hepatol.

[83] Min L, He B, Hui L (2011). Mitogen-activated protein kinases in hepatocellular carcinoma development. Seminars in cancer biology.

[84] Park GB, Choi Y, Kim YS, Lee H-K, Kim D, Hur DY (2014). ROS-mediated JNK/p38-MAPK activation regulates Bax translocation in Sorafenib-induced apoptosis of EBV-transformed B cells. Int J Oncol.

[85] Sun Z, Wang S, Zhao RC (2014). The roles of mesenchymal stem cells in tumor inflammatory microenvironment. J Hematol Oncol.

[86] Arango-Rodriguez ML, Ezquer F, Ezquer M, Conget P (2015). Could cancer and infection be adverse effects of mesenchymal stromal cell therapy?. World J Stem Cells.

[87] Maia J, Caja S, Strano Moraes MC, Couto N, Costa-Silva B (2018). Exosome-based cell-cell communication in the tumor microenvironment. Front Cell Dev Biol.

[88] Prockop DJ, Oh JY (2012). Mesenchymal stem/stromal cells (MSCs): role as guardians of inflammation. Mol Ther.

[89] Lin L, Du L (2018). The role of secreted factors in stem cells-mediated immune regulation. Cell Immunol.

[90] Davis JR, Tapon NJD (2019). Hippo signalling during development. Development.

[91] Yu F-X, Guan K (2013). The Hippo pathway: regulators and regulations. Genes Dev.

[92] Manmadhan S, Ehmer U (2019). Hippo signaling in the liver—a long and ever-expanding story. Front Cell Dev Biol.

[93] Johnson RL (2019). Hippo signaling and epithelial cell plasticity in mammalian liver development, homeostasis, injury and disease. Sci China Life Sci.

[94] Rawat SJ, Chernoff J (2015). Regulation of mammalian Ste20 (Mst) kinases. Trends Biochem Sci.

[95] Zheng T, Wang J, Jiang H, Liu LJ (2011). Hippo signaling in oval cells and hepatocarcinogenesis. Cancer Lett.

[96] Jie L, Fan W, Weiqi D, Yingqun Z, Ling X, Miao S (2013). The hippo-yes association protein pathway in liver cancer. Gastroenterol Res Pract.

[97] Zhao B, Wei X, Li W, Udan RS, Yang Q, Kim J (2007). Inactivation of YAP oncoprotein by the Hippo pathway is involved in cell contact inhibition and tissue growth control. Genes Dev.

[98] Xu MZ, Yao TJ, Lee NP, Ng IO, Chan YT, Zender L (2009). Yes-associated protein is an independent prognostic marker in hepatocellular carcinoma. Cancer.

[99] Chan SW, Lim CJ, Guo K, Ng CP, Lee I, Hunziker W (2008). A role for TAZ in migration, invasion, and tumorigenesis of breast cancer cells. Cancer Res.

[100] Xiao H, Jiang N, Zhou B, Liu Q, Du CJ (2015). TAZ regulates cell proliferation and epithelial–mesenchymal transition of human hepatocellular carcinoma. Cancer Sci.

[101] Lei QY, Zhang H, Zhao B, Zha ZY, Bai F, Pei XH (2008). TAZ promotes cell proliferation and epithelial-mesenchymal transition and is inhibited by the hippo pathway. Mol Cell Biol.

[102] Liu Y, Ren H, Wang J, Yang F, Li J, Zhou Y (2019). Prostaglandin E2 secreted by mesenchymal stem cells protects against acute liver failure via enhancing hepatocyte proliferation. FASEB J.

[103] Liu Y, Ren H, Zhou Y, Shang L, Zhang Y, Yang F (2019). The hypoxia conditioned mesenchymal stem cells promote hepatocellular carcinoma progression through YAP mediated lipogenesis reprogramming. J Exp Clin Cancer Res.

[104] Yuan F, Zhou W, Zou C, Zhang Z, Hu H, Dai Z (2010). Expression of Oct4 in HCC and modulation to wnt/β-catenin and TGF-β signal pathways. Mol Cell Biochem.

[105] El Asmar MF, Atta HM, Mahfouz S, Fouad HH, Roshdy NK, Rashed LA (2011). Efficacy of mesenchymal stem cells in suppression of hepatocarcinorigenesis in rats: possible role of Wnt signaling. J Exp Clin Cancer Res.

[106] Zhang C-L, Huang T, Wu B-L, He W-X, Liu D (2017). Stem cells in cancer therapy: opportunities and challenges. Oncotarget..

[107] Gomes CMF (2013). The dual role of mesenchymal stem cells in tumor progression. Stem Cell Res Therapy.

[108] Ma Y, Hao X, Zhang S, Zhang J (2012). The in vitro and in vivo effects of human umbilical cord mesenchymal stem cells on the growth of breast cancer cells. Breast Cancer Res Treat.

[109] Zhang Y, Wang J, Wu D, Li M, Zhao F, Ren M (2018). il-21-secreting hUcMscs combined with mir-200c inhibit tumor growth and metastasis via repression of Wnt/β-catenin signaling and epithelial–mesenchymal transition in epithelial ovarian cancer. OncoTargets Therapy.

[110] Subramanian A, Shu-Uin G, Kae-Siang N, Gauthaman K, Biswas A, Choolani M (2012). Human umbilical cord wharton’s jelly mesenchymal stem cells do not transform to tumor-associated fibroblasts in the presence of breast and ovarian cancer cells unlike bone marrow mesenchymal stem cells. J Cell Biochem.

[111] Ohlsson LB, Varas L, Kjellman C, Edvardsen K, Lindvall M (2003). Mesenchymal progenitor cell-mediated inhibition of tumor growth in vivo and in vitro in gelatin matrix. Exp Mol Pathol.

[112] Otsu K, Das S, Houser SD, Quadri SK, Bhattacharya S, Bhattacharya J (2009). Concentration-dependent inhibition of angiogenesis by mesenchymal stem cells. Blood.

[113] Lu Y, Yuan Y, Wang X, Wei L, Chen Y, Cong C (2008). The growth inhibitory effect of mesenchymal stem cells on tumor cells in vitro and in vivo. Cancer Biol Ther.

[114] Wang ML, Pan CM, Chiou SH, Chen WH, Chang HY, Lee OKS (2012). Oncostatin M modulates the mesenchymal–epithelial transition of lung adenocarcinoma cells by a mesenchymal stem cell-mediated paracrine effect. Cancer Res.

[115] Ryu H, Oh J-E, Rhee K-J, Baik SK, Kim J, Kang SJ (2014). Adipose tissue-derived mesenchymal stem cells cultured at high density express IFN-β and suppress the growth of MCF-7 human breast cancer cells. Cancer Lett.

[116] Du J, Zhou L, Chen X, Yan S, Ke M, Lu X (2012). IFN-γ-primed human bone marrow mesenchymal stem cells induce tumor cell apoptosis in vitro via tumor necrosis factor-related apoptosis-inducing ligand. Int J Biochem Cell Biol.

[117] Kolluri KK, Laurent GJ, Janes SM (2013). Mesenchymal stem cells as vectors for lung cancer therapy. Respiration..

[118] Loebinger M, Sage E, Davies D (2010). Janes SJBjoc. TRAIL-expressing mesenchymal stem cells kill the putative cancer stem cell population..

[119] Zhao D, Hou L, Pan M, Hua J, Wang Z, He J (2018). Inhibitory effect and mechanism of mesenchymal stem cells cultured in 3D system on hepatoma cells HepG2. Appl Biochem Biotechnol.

[120] Studeny M, Marini FC, Dembinski JL, Zompetta C, Cabreira-Hansen M, Bekele BN (2004). Mesenchymal stem cells: potential precursors for tumor stroma and targeted-delivery vehicles for anticancer agents. J Natl Cancer Inst.

[121] Waterman RS, Tomchuck SL, Henkle SL, Betancourt AMJ (2010). A new mesenchymal stem cell (MSC) paradigm: polarization into a pro-inflammatory MSC1 or an Immunosuppressive MSC2 phenotype. PLoS ONE.

[122] Waterman RS, Henkle SL, Betancourt AMJ (2012). Mesenchymal stem cell 1 (MSC1)-based therapy attenuates tumor growth whereas MSC2-treatment promotes tumor growth and metastasis. PLoS ONE.

[123] Gwendal L (2016). Recent discoveries concerning the tumor-mesenchymal stem cell interactions. BBA Rev Cancer.

[124] Pan M, Hou L, Zhang J, Zhao D, Hua J, Wang Z (2018). Inhibitory effect and molecular mechanism of mesenchymal stem cells on NSCLC cells. Mol Cell Biochem.

[125] Lou G, Song X, Yang F, Wu S, Wang J, Chen Z (2015). Exosomes derived from miR-122-modified adipose tissue-derived MSCs increase chemosensitivity of hepatocellular carcinoma. J Hematol Oncol.

[126] Kean TJ, Lin P, Caplan AI, Dennis JE (2013). MSCs: delivery routes and engraftment, cell-targeting strategies, and immune modulation. Stem Cells Int.

[127] Hu Y-L, Fu Y-H, Tabata Y, Gao J-Q (2010). Mesenchymal stem cells: a promising targeted-delivery vehicle in cancer gene therapy. J Control Release.

[128] Zhou J, Tan X, Tan Y, Li Q, Ma J, Wang G (2018). Mesenchymal stem cell derived exosomes in cancer progression, metastasis and drug delivery: a comprehensive review. J Cancer..

[129] Goto T, Murata M, Terakura S, Nishida T, Adachi Y, Ushijima Y (2018). Phase I study of cord blood transplantation with intrabone marrow injection of mesenchymal stem cells: a clinical study protocol. Medicine..

[130] Fernández O, Izquierdo G, Fernández V, Leyva L, Reyes V, Guerrero M (2018). Adipose-derived mesenchymal stem cells (AdMSC) for the treatment of secondary-progressive multiple sclerosis: a triple blinded, placebo controlled, randomized phase I/II safety and feasibility study. PLoS ONE.

[131] Al-Najar M, Khalil H, Al-Ajlouni J, Al-Antary E, Hamdan M, Rahmeh R (2017). Intra-articular injection of expanded autologous bone marrow mesenchymal cells in moderate and severe knee osteoarthritis is safe: a phase I/II study. J Orthop Surg Res.

[132] Li GC, Ye QH, Xue YH, Sun HJ, Zhou HJ, Ren N (2010). Human mesenchymal stem cells inhibit metastasis of a hepatocellular carcinoma model using the MHCC97-H cell line. Cancer Sci.

[133] Zhao W, Ren G, Zhang L, Zhang Z, Liu J, Kuang P (2012). Efficacy of mesenchymal stem cells derived from human adipose tissue in inhibition of hepatocellular carcinoma cells in vitro. Cancer Biother Radiopharm.

[134] Li T, Song B, Du X, Wei Z (2013). Huo TJEjomr. Effect of bone-marrow-derived mesenchymal stem cells on high-potential hepatocellular carcinoma in mouse models: an intervention study..

[135] Qiao L, Xu Z, Zhao T, Zhao Z, Shi M, Zhao RC (2008). Suppression of tumorigenesis by human mesenchymal stem cells in a hepatoma model. Cell Res.

[136] Bruno S, Collino F, Deregibus MC, Grange C, Tetta C, Camussi GJ (2012). Microvesicles derived from human bone marrow mesenchymal stem cells inhibit tumor growth. Stem Cells Dev.

[137] Ma B, Jiang H, Jia J, Di L, Song G, Yu J (2012). Murine bone marrow stromal cells pulsed with homologous tumor-derived exosomes inhibit proliferation of liver cancer cells. Clin Transl Oncol.

[138] Abd-Allah SH, Shalaby SM, Amal S, Elkader EA, Hussein S, Emam E (2014). Effect of bone marrow–derived mesenchymal stromal cells on hepatoma. Cytotherapy..

[139] Seyhoun I, Hajighasemlou S, Muhammadnejad S, Ai J, Nikbakht M, Alizadeh A (2018). Combination therapy of sorafenib with mesenchymal stem cells as a novel cancer treatment regimen in xenograft models of hepatocellular carcinoma. J Cell Physiol.

[140] Hajighasemlou S, Pakzad S, Ai J, Muhammadnejad S, Mirmoghtadaei M, Hosseinzadeh F (2018). Characterization and Validation of hepatocellular carcinoma (HCC) xenograft tumor as a suitable liver cancer model for preclinical mesenchymal stem cell studies. APJCP.

[141] Hernanda PY, Pedroza-Gonzalez A, van der Laan LJ, Bröker ME, Hoogduijn MJ, Ijzermans JN (2013). Tumor promotion through the mesenchymal stem cell compartment in human hepatocellular carcinoma. Carcinogenesis.

[142] Yan XL, Jia YL, Chen L, Zeng Q, Zhou JN, Fu CJ (2013). Hepatocellular carcinoma-associated mesenchymal stem cells promote hepatocarcinoma progression: role of the S100A4-miR155-SOCS1-MMP9 axis. Hepatology.

[143] Gong P, Wang Y, Wang Y, Jin S, Luo H, Zhang J (2013). Effect of bone marrow mesenchymal stem cells on hepatocellular carcinoma in microcirculation. Tumor Biol.

[144] Jing Y, Han Z, Liu Y, Sun K, Zhang S, Jiang G (2012). Mesenchymal stem cells in inflammation microenvironment accelerates hepatocellular carcinoma metastasis by inducing epithelial-mesenchymal transition. PLoS ONE.

[145] Bhattacharya SD, Mi Z, Talbot LJ, Guo H, Kuo PCJS (2012). Human mesenchymal stem cell and epithelial hepatic carcinoma cell lines in admixture: concurrent stimulation of cancer-associated fibroblasts and epithelial-to-mesenchymal transition markers. Surgery.

[146] Liu C, Liu Y, Xu X, Guo X, Sun G, Ma X (2016). Mesenchymal stem cells enhance the metastasis of 3D-cultured hepatocellular carcinoma cells. BMC Cancer.

